# Vitamin C-induced oxalate nephropathy in a renal transplant patient related to excessive ingestion of cashew pseudofruit (Anacardium occidentale L.): a case report

**DOI:** 10.1186/s12882-018-1060-9

**Published:** 2018-10-12

**Authors:** Miguel Moyses-Neto, Bruno Rafael Santos Brito, Dyego José de Araújo Brito, Noelia Dias Carneiro Barros, Márcio Dantas, Natalino Salgado-Filho, Roberto Silva Costa, Gyl Eanes Barros Silva

**Affiliations:** 1 Nephrology Division, Department of Internal Medicine, Ribeirão Preto Medical School, University of São Paulo, Av. Bandeirantes, 3900 - Monte Alegre, Ribeirão Preto, SP 14049-900 Brazil; 2 Kidney Disease Prevention Centre and University Hospital, Federal University of Maranhão, Barão de Itapary Street, 227, Centro, São Luís, MA 65020-070 Brazil; 3 Department of Pathology, Ribeirão Preto Medical School, University of São Paulo, Av. Bandeirantes, 3900 - Monte Alegre, Ribeirão Preto, SP 14049-900 Brazil

**Keywords:** Ascorbic acid, Cashew pseudofruit, Renal function, Dialysis, Acute kidney injury, Oxalate nephropathy, Vitamin C, Renal transplant, Acute renal injury, Cashew

## Abstract

**Background:**

Ingestion of vitamin C is generally regarded as harmless. Oxalate nephropathy is an infrequent condition and is characterized by oxalate deposition in the renal tubules, in some cases resulting in acute kidney injury. It can be caused by overproduction of oxalate in genetic disorders and, more frequently, as a secondary phenomenon provoked by ingestion of oxalate or substances that can be transformed into oxalate in the patient.

**Case presentation:**

We present a case of acute oxalate nephropathy in a 59-year-old black male with type 2 diabetes mellitus, who received a kidney transplant 11 years prior. He ingested a large amount of cashew pseudofruit (“cashew apple”) during 1 month and developed acute kidney injury. His previous blood creatinine was 2.0 mg/dL, which increased to 7.2 mg/d; he required hemodialysis. He was subsequently discharged without need for dialysis; 3 months later his blood creatinine stabilized at 3.6 mg/dL.

**Conclusions:**

This pseudofruit is rich in ascorbic acid (vitamin C) and poor in oxalate. Urinary oxalate excretion begins to increase when amounts of ascorbic acid above bodily requirements are ingested, and may provoke acute oxalate nephropathy. The patient’s oxalate acute nephropathy, in this case, was attributed to excessive vitamin C ingestion from the cashew pseudofruit associated with decreased renal function.

## Background

**Fig. 1:**
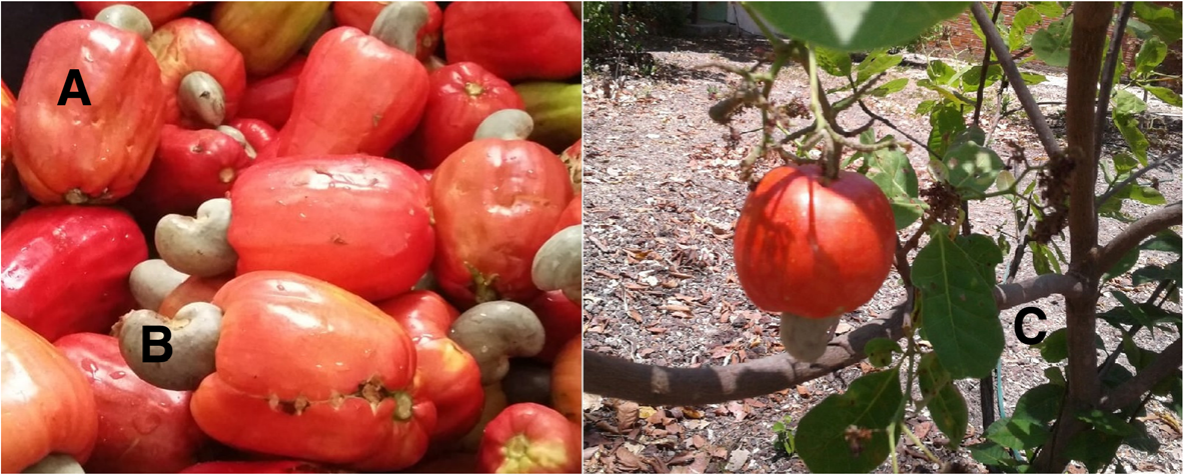
Cashew. **a** Cashew pseudofruit; (**b**) Fruit (cashew nut); (**c**) Cashew tree. Images are the authors’ own

Cashew is known as the cashew tree fruit (Anacardium occidentale L.), but it is actually a pseudofruit (“cashew apple”). The real fruit is the cashew nut. It is believed to have originated in Brazil, but is has been cultivated in southeast Asia and also in some countries in Africa [1]. In Brazil, it is served as a fresh beverage or in natura. The pseudofruit is very rich in vitamin C (219.3 mg/100 g) [2] but poor in oxalate (Fig. 1). The nut, instead, is rich in oxalate [3]. Secondary oxalosis and oxalate nephropathy can occur in primary or secondary hyperoxaluria. Secondary hyperoxaluria may be due to increased dietary intake of oxalate as seen with star fruit ingestion [4], cashew nut ingestion [5], increased absorption from the bowel (enteric hyperoxaluria) [6], and increased production of oxalate, especially caused by increased levels of oxalate precursors, commonly glyoxalate associated with ethylene glycol ingestion and ascorbic acid [7]. We present a case of a renal transplant patient who ingested a large amount of ascorbic acid due to excessive ingestion of cashew pseudofruit and juice.

## Case presentation

**Fig. 2:**
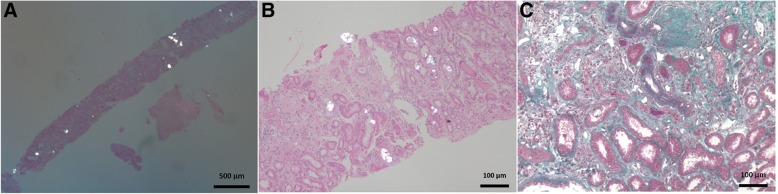
Kidney biopsy showing extensive birefringent calcium oxalate crystal deposition and epithelial degenerative changes. **a** and **b**. Polarized microscopy (hematoxylin and eosin) in a background of interstitial fibrosis, tubular atrophy, glomerulosclerosis, and hyaline arteriosclerosis. **c** Masson trichrome.

A 59-year-old black male with type 2 diabetes mellitus progressed to end stage renal failure; he received a kidney from his wife 11 years prior to presentation. He came to the outpatient clinic for routine screening and was asymptomatic. Maintenance immunosuppression therapy included tacrolimus, prednisone, and azathioprine. He was also taking medications for hypertension (atenolol and enalapril), dyslipidemia (atorvastatin), hypothyroidism (levothyroxine), and diabetes (insulin NPH). Surprisingly, he was found to have a blood creatinine level of 7.2 mg/dL and blood urea of 81 mg/dL. His previously blood creatinine levels were 2.0 mg/dL 2 months prior and in the range of 2.0 to 2.5 mg/dL 4 months prior, estimated glomerular filtration rate (GFR) by MDRD (Modification of Diet in Renal Disease Study equation): 39.6 mL/min/1.73 m2). Other laboratory results were as follows: hemoglobin (Hb), 12.0 g/L; sodium (Na), 145 mEq/L; potassium (K), 4.3 mEq/L; uric acid, 10.5 mg/dL; aspartate aminotransferase (AST), 14 U/L; alanine transaminase (ALT), 13 U/L; total calcium, 8.3 mg/dL; bicarbonate, 16.6 mE/L; albumin, 3.4 g/dL. The patient did not smoke or drink alcohol and denied any type of surgery. He also denied recent episodes of diarrhea or antibiotic use. On physical examination, he had a blood pressure of 130/80 mmHg, pulse rate of 68 beats/min, respiratory rate of 16/min, and weight of 54 kg. He had no signs of edema or dehydration. The patient was admitted to the hospital and underwent hemodialysis. A kidney biopsy was performed. Histologic analysis showed cortical and medullary areas with nine glomeruli, one of which was sclerotic, and two arteries. The pathology report of the kidney biopsy was compatible with oxalate nephropathy and severe acute tubular necrosis associated with intense calcium oxalate deposition (Fig. 2). There were no signs of rejection and C4d immunohistochemistry was negative. Fibrosis of the interstitium was moderate. Two days after the biopsy, urine sample was collected and centrifuged; calcium oxalate crystals could be seen under polarized light. The patient denied ingestion of products containing ethylene glycol, any other medication such as orlistat, and any other drugs different from what was prescribed to him. However, he reported that in the last month he had been eating 5 cashew pseudofruit (“cashew apple”) every day and drinking a large amount of cashew pseudofruit juice (about 1000 mL) every day instead drinking water. The fruits were picked up from a tree in the patient’s backyard. He did not eat cashew nuts. We could deduce that the patient was ingesting every day, for at least 1 month, approximately 2 to 3 g of ascorbic acid in his diet. This calculation was based on an ascorbic acid concentration of 219.3 mg/100 mL in the pseudofruit. The patient underwent five hemodialysis sessions and was discharged with a creatinine of 3.9 mg/dL and no further requirement for dialysis. Three months after hospital discharge, his creatinine was 3.4 mg/dL (MDRD GFR: 24 mL/min/1.732).

## Discussion and conclusions

The most common causes of hyperoxaluria and oxalate nephropathy are primary hyperoxaluria type I and II (intrinsic overproduction of oxalate), secondary oxalosis due to dietary oxaluria after ingestion of certain foods such as star fruit and cashew nuts [5, 6], enteric hyperoxaluria caused by malabsorption (as seen with jejunoileal bypass and small bowel resection for Crohn disease), gastric and pancreatic lipase inhibitor use (e.g. orlistat), increased oxalate excretion after renal transplantation, and increased ingestion of oxalate precursors such as ethylene glycol and vitamin C [6]. Other associated causes include chronic pancreatitis, diabetic gastroenteropathy, prolonged antibiotic therapy, and chronic kidney disease [6]. In the present case, the patient did not have hyperoxaluria types I and II, did not have enteric hyperoxaluria or gastrointestinal malabsorption disease, and had no history of ethylene glycol ingestion, use of foods rich in oxalate, or prolonged antibiotic therapy.

Oxalate is eliminated almost exclusively by the kidneys and is readily filtered through the glomerulus and excreted in the proximal tubules [6]. In cases of acute or chronic excessive hyperoxaluria, it can crystallize in the tubular lumen, injure the tubular epithelium, and obstruct the tubular lumen [4].

In renal failure, oxalate excretion decreases roughly in proportion to the decrease in renal function as serum oxalate concentration increases [6, 8]. The blood levels of oxalate can achieve supersaturation and precipitate mainly in the kidneys, bones, joints, cardiac conductive system, blood vessels, and retina [9].

After successful renal transplantation, excess plasma oxalate is cleared, resulting in transient hyperoxaluria lasting from 3 days to 3 weeks [10]. In some patients, this deposition of calcium oxalate occurs more frequently in the first year post surgery and is associated with a possible negative impact on graft function beyond the early post-transplant period, provoking early acute kidney injury or as an additional non-immunologic factor that will impact the graft later [10–12]. In the present case the patient underwent renal transplant 11 years prior.

Ascorbic acid, also known as vitamin C, should be replaced each day by dietary intake of 70 mg to 90 mg to maintain optimal health and ascorbic acid homeostasis [7]. Urinary oxalate excretion begins to increase when amounts of ascorbic acid above that required by the body are ingested [7]. There are many reports of oxalate nephropathy associated with moderate and large amounts of oral and intravenous administration of vitamin C in people with previously normal renal function [13–17]. In another report, the authors describe fatal vitamin C-associated acute renal failure, without reported the patient’s previous renal function [18]. In a short-term human experiment, Auer et al. [19] described a 25-year-old individual, with no history of nephrolithiasis and normal renal function, who ingested 8 g of ascorbic acid during 8 days. After 8 days, he presented with hematuria after oxalate excretion had increased to 350%, showing crystalluria, and the protocol was immediately suspended. Although the authors highlighted the potential dangers of large dose ingestion of vitamin C in some individuals, they did not show alterations in renal function.

Recently, some reports of patients with renal allografts developing oxalate nephropathy and worsening of renal function with vitamin C ingestion have been reported [20, 21]. Getting JE et al. [20] described 65 patients with biopsy proven calcium oxalate crystals. Among these patients, five patients showed oxalate nephropathy associated with high intake of vitamin C, including two patients status post kidney transplant and three patients with chronic kidney diseaseysli. Suneja M et al. [21] described three patients, two with a history of kidney transplant and one with a history of pancreas-kidney transplant. All three patients had history of vitamin C ingestion. They presented with acute kidney allograft dysfunction and oxalate nephropathy on renal biopsies. We do not know when the transplants occurred in these patients.

In the present case, 11 years after kidney transplant, the patient had chronic renal disease with reduced creatinine clearance, as demonstrated on renal biopsy. He also had high ingestion of vitamin C related to the ingestion of cashew pseudofruit. Oxalate nephropathy is more likely to develop in patients with more than one predisposing factor. The patient’s oxalate acute nephropathy was attributed to excessive vitamin C ingestion associated with renal dysfunction, since the oxalate content in this pseudofruit is very low, although the oxalate content in the nut is high [3]. To our knowledge, this is the first reported case of oxalate nephropathy provoked by vitamin C due to cashew pseudofruit ingestion. Vitamin C is prescribed for a number of indications, and clinicians should be aware of the potential risks of high doses of vitamin C ingestion and the ingestion of large amount of fruits rich in vitamin C, especially for patients with renal transplant or other nephropathies and decreased renal function.

## Abbreviations

ALT: Alanine transaminase
AST: Aspartate aminotransferase
Hb: Hemoglobin
K: Potassium
MDRD GFR: Modification of Diet in Renal Disease Study equation for estimated glomerular filtration rate
Na: Sodium

## References

[1] Lima AC, Garcia NHP, Lima JR (2004). Obtenção e caracterização dos principais produtos do caju. Boletim CEPPA.

[2] NEPA/UNICAMP - Núcleo de Estudos e Pesquisas em Alimentação/ Universidade de Campinas (2011). TACO - Tabela brasileira de composição de alimentos.4.

[3] Massey LK (2007). Food oxalate: factors affecting measurement, biological variation, and bioavailability. J Am Diet Assoc.

[4] MoysesNeto M, Silva GEB, Costa RS, Vieira-Neto OM, Garcia-Cairasco N, Lopes NP, Haendchen PF, Silveira C, Mendes AR, Filho RR, Dantas M (2009). Star fruit: simultaneous neurotoxic and nephrotoxic effects in people with previously normal renal function. NDT Plus.

[5] Bernardino Margarida, Parmar Malvinder S. (2016). Oxalate nephropathy from cashew nut intake. Canadian Medical Association Journal.

[6] Glew R, Sun Y, Horowitz BL, Konstantinov KN, Barry M, Fair JR, Masssie L, Tzamaloukas H (2014). Nephropathy in dietary hyperoxaluria: a potentially preventable acute or chronic kidney disease. World J Nephrol.

[7] Knight J, Madduma-Liyanage K, Mobley JA, Assimos DG, Holmes RP (2016). Ascorbic acid intake and oxalate synthesis. Urolithiasis.

[8] Liu Y, Weisberg LS, Langman CB, Logan A, Hunter K, Prasad D, Avila J, Venkatchalan T, Berns JS, Handelman GJ, Sirovere WD (2016). Plasma oxalate levels in prevalent hemodialysis patients and potential implications for ascorbic acid supplementation. ClinBiochem.

[9] Worcester EM, Nakagawa Y, Bushinsky DA, Coe FL (1986). Evidence that serum calcium oxalate supersaturation is a consequence of oxalate retention in patients with chronic renal failure. J Clin Invest.

[10] Bagnasco SM, Mohammed BS, Mani H, Gandolfo MT, Haas M, Racusen LC, Montgomery RA, Kraus E (2009). Oxalate deposits in biopsies from native and transplanted kidneys, and impact on graft function. Nephrol Dial Transpl.

[11] Truong LD, Yakupoglu U, Feig D, Hicks J, Cartwight J, Sheikh-Hamad D, Suki WN (2004). Calcium oxalate deposition in renal allografts: morphologic spectrum and clinical implications. Am J Transpl.

[12] Pinheiro HS, Camara NOS, Osaki KS, De Moura LAR, Pacheco-Silva A (2005). Early presence of calcium oxalate deposition in kidney graft biopsies is associated with poor long-term graft survival. Am J Transpl.

[13] Mashour S, Turner JF, Merrel R (2000). Acute renal failure, oxalosis, and vitamin C supplementation, a case report and review of the literature. Chest.

[14] Nasr SH, Kashtanova Y, Levchuk V, Markowitz GS (2006). Secondary oxalosis due to excess vitamin C intake. Kidney Int.

[15] Lamarche J, Nair R, Peguero A, Courville C (2011). Vitamin C-induced oxalate nephropathy. Int J Nephrol.

[16] Gurm H, Sheta MA, Nivera N, Tunkel A (2012). Vitamin-C inducedoxalatenephropathy. J Community HospInt Med Persp.

[17] Cossey LN, Rahim F, Larsen CP (2013). Oxalate nephropathy and intravenous vitamin C. Am J Kidney Dis.

[18] GJM H, Graber ML, Freebairn RC (2008). Fatal vitamin C-associated acute renal failure. Anaesth Intensive Care.

[19] Auer BL, Auer D, Rodgers AL (1998). Relative hyperoxaluria, crystalluria and haematuria after megadose ingestion of vitamin C. Eur J Clin Investig.

[20] Getting JE, Gregoire JR, Phul A, Kasten MJ (2013). Oxalate nephropathy due to “juicing”: case report and review. Am J Med.

[21] Suneja M, Kumar AB (2013). Secondary oxalosis induced acute kidney injury in allograft kidneys. Clin Kidney J.

